# Abemaciclib and Letrozole in Metastatic Male Breast Cancer

**DOI:** 10.1002/cnr2.70054

**Published:** 2024-11-15

**Authors:** Leon Schönfeld, Christian Möhring, Rouven Strobel, Hanns Leonhard Kaatsch, Stephan Waldeck, Ulrike Wagner

**Affiliations:** ^1^ Department for Internal Medicine Bundeswehr Central Hospital Koblenz Germany; ^2^ Department for Diagnostic and Interventional Radiology and Neuroradiology Bundeswehr Central Hospital Koblenz Germany

**Keywords:** abemaciclib, CDK4/6 inhibitor, male breast cancer, metastatic disease

## Abstract

**Background:**

Male breast cancer is a very rare disease and only accounts for around 1% of all breast cancers. The treatment strategies are based on those used for breast cancer in women. So far, there is a lack of randomized data to support specific treatment modalities in men. To our knowledge, a therapeutic approach with a combination of letrozole and abemaciclib has not yet been described for a male patient with metastatic breast cancer.

**Case Description:**

Here, we report a case of a male patient with advanced metastatic breast cancer treated with a combination of letrozole and abemaciclib. The therapy was well tolerated with no reported side effects. The follow‐up CT showed regression of the primary tumor mass and the lymph nodes and soft tissue metastases.

**Conclusion:**

In summary, this case report clearly shows the effectiveness of the therapeutic approach and should be discussed for the treatment of future patients.

## 1 Background

Male breast cancer (MBC) is a very rare condition, accounting for only about 1% of all breast cancers. Often, the diagnosis is made at an advanced stage, resulting in increased mortality [1]. The 5‐ and 10‐year survival rates for MBC are reported to be 77% and 68%, respectively [2]. Risk factors for developing MBC include a shift in the androgen/estrogen ratio due to obesity, alcoholism, liver cirrhosis, and testicular dysfunction. Another risk factor is a positive family history, with first‐degree relatives having a threefold increased risk for MBC. Genetic risk mutations are associated with homologous recombination deficiency. About 10% of patients have a BRCA‐2 mutation [3, 4]. Clinically, the disease most commonly presents as tumor growth, nipple retraction, or secretion. The diagnosis is made a median of 6 months after the onset of symptoms. Due to the often delayed diagnosis, up to 31% of patients have local lymph node involvement at the time of initial diagnosis [3, 5]. The most common entity of MBC is invasive ductal carcinoma. Most carcinomas are hormone receptor‐positive. HER2‐neu positivity is seen in a few cases. The Ki67 labeling index, a proliferation marker, is described as having an activity of < 20% in 75% of cases. The androgen receptor is positive in 96.9% of cases [6]. Prognostically, male patients show significantly higher mortality compared to female breast cancer patients (FBC). The 3‐year mortality for male patients is 15% higher, and the 5‐year mortality is 19% higher than for female patients [7].

Various therapeutic regimens exist for metastatic MBC, modeled after the treatment of female breast cancer, as prospective randomized studies on MBC are lacking. First‐line therapy for metastatic, hormone receptor‐positive carcinoma is recommended to be Tamoxifen. This therapy can be supplemented with an aromatase inhibitor and/or a gonadotropin‐releasing hormone analogue and/or the estrogen receptor antagonist fulvestrant. Furthermore, some guidelines also recommend a cyclin‐dependent kinase inhibitors combined with an aromatase inhibitor as first‐line options, as this combination has shown very good responses in FBC. Chemotherapy may be considered for hormone receptor‐negative patients [3, 8].

Cyclin‐dependent kinase inhibitors have already been established for FBC in the MONARCH 3, PALOMA 2, and MONALEESA 2 and 7 trials. For MBC patients, data is limited. The POLARIS study by Blum et al. and the multicenter study by Yildirim et al. emphasize the efficacy of cyclin‐dependent kinase inhibitors. Abemaciclib was not included in these studies. Regarding the efficacy of abemaciclib, there is a case series and case reports with very good response rates [8, 9, 10, 11, 12, 13, 14].

Biologically, cyclin‐dependent kinase inhibitors work synergistically with anti‐estrogens to induce a G1 cell cycle arrest. Estrogen promotes CDK4/6 activity and leads to inhibition of the retinoblastoma protein through hyperphosphorylation, resulting in progression in the cell cycle. By inhibiting both estrogen and CDK4/6 activity, this progression is prevented, halting the cell cycle [15, 16, 17].

MBC is a very rare cancer and standard treatment in the metastatic stage is provided by data extrapolated from studies conducted on female patients. This case report adds important data for the therapy of future patients.

## 2 Case

The patient was presented to our interdisciplinary emergency room in the German army hospital in Koblenz on June 5, 2023 with tumorous growth of the right breast that had been progressive for 3 years.

For 2 months, he had also noticed ulcerations with repeated bleeding.

Upon admission, he reported no weight loss, night sweats, fever, chills, or severe pain. The examination revealed a reddish‐purple exophytic, non‐mobile tumor ~15 × 15 cm in diameter in the right pectoral area with central ulceration. There was a deep wound cavity about 5 cm in diameter with clear exudate. Multiple reddish indurations, up to 5 cm in size, were present over the anterior chest wall, indicating satellite lesions. Enlarged axillary and cervical lymph nodes were palpable along the sternocleidomastoid muscle, some of which were non‐mobile. The patient was admitted to our oncology ward for biopsy and staging with an initial suspicion of Merkel cell carcinoma. Laboratory findings were unremarkable. The patient was 65 years old at the time of presentation in our outpatient clinic, with a body mass index of 30. There was no prior history of smoking or consuming alcohol. Past medical history included arterial hypertension and pectus excavatum surgery at age 11. There were no known allergies, and the patient was not on any regular medication at the time of admission. A family history of bone cancer of unclear origin was reported on the paternal side.

### 2.1 Diagnostic Workup

**FIGURE 1:**
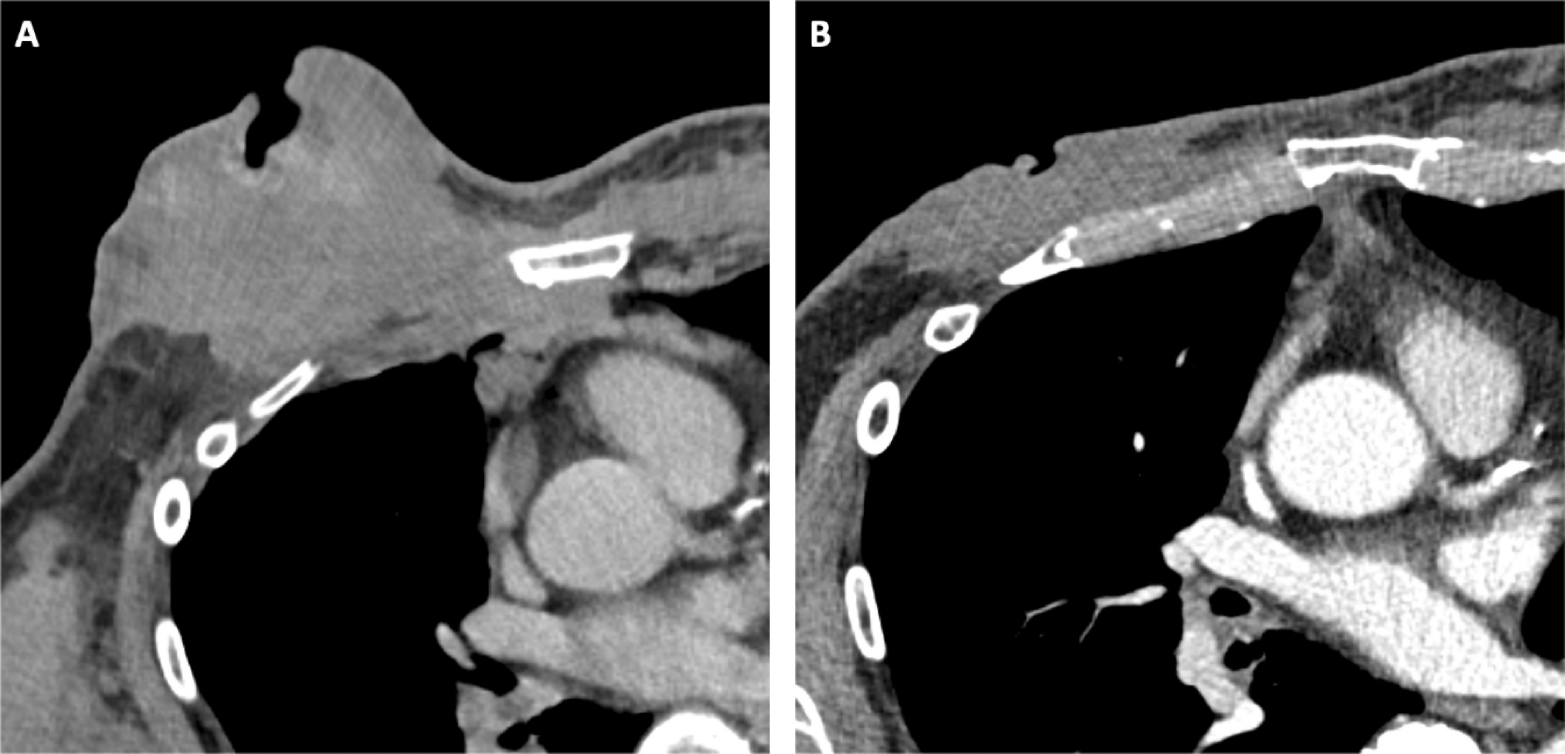
Contrast‐enhanced CT scans of the thorax in the axial soft tissue window before (A) and 6 months after the start of therapy (B). There is a large ulcerating soft tissue tumor in the right mammillary area extending sternally with infiltration of the right pectoralis major muscle, the chest wall and the mediastinum (A). The follow‐up CT showed a clear size reduction of the right mammillary tumor mass as well as chest wall and mediastinal infiltration as a morphological correlate of the clinical response to therapy.

Further staging by PET CT on June 6, 2023, showed an extensive ulcerating glucose hypermetabolic mass on the right pectoral side with infiltration into the chest/shoulder/neck muscles with accompanying lymphogenic metastasis cervically, mediastinally, axillary, and retroperitoneally (Figure 1). Preliminary histopathological findings confirmed intradermal metastasis of Merkel cell carcinoma. We initiated therapy with Avelumab 800 mg on June 16, 2023, suspecting metastatic Merkel cell carcinoma. After starting therapy, an additional histopathological report diagnosed a poorly differentiated ductal carcinoma with lymphovascular invasion. We diagnosed a multiple lymphatic and soft tissue metastasized invasive ductal carcinoma of the right breast. The tumor was classified as T4b, L1, G3, and N1, corresponding to UICC Stage 4. The tumor showed strong estrogen receptor expression (IRS = 12), no progesterone receptor expression, a high proliferation index (Ki67 = 30%), no HER2/neu overexpression, and no PD‐L1 expression. A BRCA analysis was not performed. At this point, we recommended a transferal to a breast cancer center. However, the patient wished to continue therapy at our oncology clinic.

### 2.2 Medical Management and Treatment

We adjusted the therapy according to the new diagnosis and established treatment with the aromatase inhibitor Letrozole 2.5 mg once daily and Abemaciclib 150 mg twice daily on June 26, 2023. After initiating the therapy, follow‐up examinations showed good tolerability. The patient did not report any side effects, for example, nausea, loss of appetite, fatigue, infections, or thrombosis. In the first follow‐up, the patient described a better and softer feeling in the area of skin metastasis. The ulcerated tumor area was overall more vulnerable with an increased tendency to bleed. Subsequent clinical follow‐ups showed a very good therapeutic response.

**FIGURE 2:**
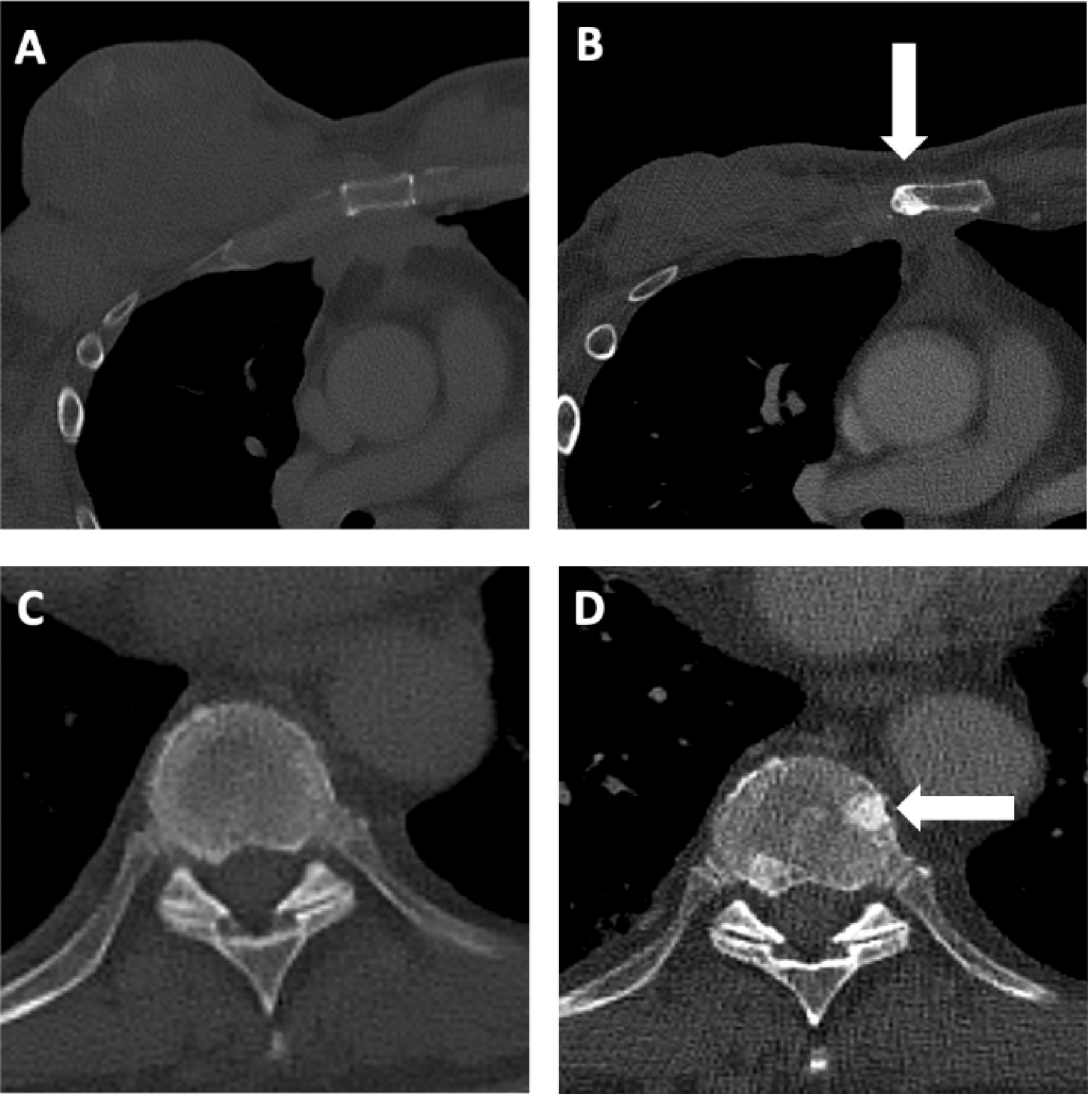
Corresponding to Figure 1, axial bone window images of the identical thoracic CT before (A, C) and 6 months after the initiation of therapy (B, D) are shown. During follow‐up newly defined focal sclerotic bone lesions (arrows) were identified in the corpus sterni on the right adjacent to the sternum and in the ninth thoracic vertebra on the left. In view of the clear size regression of the soft tissue tumor and the lack of evidence of progressive bone metastases, these lesions were classified as an osteoblastic reaction in the course of therapeutic response of sternal infiltration by the soft tissue tumor and previously occult vertebral body metastasis.

The clinical findings were then confirmed in the follow‐up CT of the neck and thorax on January 25, 2024. The primary tumor was clearly reduced in size, as were the lymph node and right thoracic soft tissue metastases. In addition, newly defined focal sclerotic bone lesions (Figure 2) in the corpus sterni and in the ninth thoracic vertebra were identified. In view of the distinct size regression of the tumor mass and absence of progressive bone metastases, we classified these lesions as osteoblastic responses during a healing reaction. To prevent osteoporosis, despite the osteoblastic nature of the lesions, we began therapy with alendronate 70 mg once a week, after previous jaw necrosis had been ruled out by the oral and maxillofacial surgery department. The follow‐up CT from July 2024 showed no progression of the tumor disease. The osteoblastic lesion at the ninth thoracic vertebra also remained stable in appearance. The patient's last follow‐up visit was on September 22, 2024. During this visit, the patient was observed to be in good clinical condition. His quality of life was not impacted by the therapy. The patient continued to report no adverse events during follow‐up examinations.

## 3 Conclusion

We initiated a therapy using a cyclin‐dependent kinase inhibitor and an aromatase inhibitor. This combination is approved for the first‐line systemic treatment of metastatic breast cancer in women [8].

For MBC, there are only sporadic reports in the literature. The POLARIS study by Blum et al. reported on treatment with palbociclib as monotherapy. A complete response was achieved in 6.7%, a partial response in 26.7%, and stable disease in 53.3% of patients. The mPFS (median progression free survival) was 19.8 months [18].

The multicenter study of Yildirim et al. highlights the efficacy of palbociclib and ribociclib as first‐line treatment for metastatic hormone receptor‐positive, human epidermal growth factor receptor 2‐negative breast cancer in male patients. An ORR (overall response rate) of 84% for palbociclib and 76.2% for ribociclib were observed. The mPFS of the entire cohort was 28.06 months. No adverse events occurred. The study supports the use of cyclin‐dependent kinase inhibitors in MBC [12].

The conclusions of these studies are, of course, subject to limitations due to the sample size and retrospective nature. The studies by Yildirim et al. and Blum et al. show similar mPFS and ORR to the MONARCH‐3 and PALOMA‐2 studies. In these studies, an mPFS of 28 months and 24.8 months, respectively, and an ORR of 61% and 55% were observed [8, 10].

For MBC, there are few case reports on cyclin‐dependent kinase inhibitors. Hansra et al. reported a complete remission with abemaciclib, fulvestrant, and leuprolide [13, 14].

In comparison with the previous therapy regimen of an aromatase inhibitor in combination with a gonadotropin‐releasing hormone analogues (GnRH), the current studies show a significant improvement in mPFS. The mPFS of MBC patients who received an aromatase inhibitor with a GnRH was 10 months [19].

Due to the heterogeneous and limited data, we decided on treatment with abemaciclib and letrozole. The tumor showed an impressive response with significant remission of the tumor mass under the initiated therapy. The follow‐up CT examination also showed newly demarcated focal sclerotic lesions in the right corpus sterni adjacent to the soft tissue tumor, as well as in the left ninth thoracic vertebra. Considering the significant reduction in the size of the soft tissue tumor and the lack of evidence for further progressive bony metastasis, the depicted bone changes are consistent with an osteoblastic reaction (“osteoblastic flare”) as part of the treatment response. According to the RECIST 1.1 criteria and MD Anderson (MDA) criteria, this response is classified as a partial remission [20].

In summary, this case report demonstrates the remarkable response of an already advanced, metastatic MBC with a high tumor burden. There is limited evidence for MBC. This case highlights the effectiveness of the combination with abemaciclib and letrozole and suggests that it warrants further consideration for MBC treatment strategies. As a single case report, there is a need for larger clinical trials to validate our findings.

## Author Contributions

**Leon Schönfeld:** conceptualization, methodology, visualization, writing – review and editing, writing – original draft. **Christian Möhring:** supervision, writing – review and editing. **Rouven Strobel:** conceptualization, writing – review and editing, supervision. **Hanns Leonhard Kaatsch:** resources, supervision, writing – review and editing. **Stephan Waldeck:** writing – review and editing, resources. **Ulrike Wagner:** writing – review and editing, supervision.

## Consent

Written informed patient consent was obtained for publication of the data contained in this case report.

## Conflicts of Interest

The authors declare no conflicts of interest.

## Data Availability

The data that support the findings of this study are available on request from the corresponding author. The data are not publicly available due to privacy or ethical restrictions.

## References

[1] A. J. Abdelwahab Yousef , “Male Breast Cancer: Epidemiology and Risk Factors,” Seminars in Oncology 44, no. 4 (2017): 267–272. 29526255 10.1053/j.seminoncol.2017.11.002

[2] Robert Koch Institut Z für K , “Brustkrebs (Mammakarzinom),” accessed April 14, 2024, https://www.krebsdaten.de/Krebs/DE/Content/Krebsarten/Brustkrebs/brustkrebs_node.html.

[3] G. Zheng and J. P. Leone , “Male Breast Cancer: An Updated Review of Epidemiology, Clinicopathology, and Treatment,” Journal of Oncology 2022 (2022): 1734049. 35656339 10.1155/2022/1734049 PMC9155932

[4] M. Szwiec , J. Tomiczek‐Szwiec , W. Kluźniak , et al., “Genetic Predisposition to Male Breast Cancer in Poland,” BMC Cancer 21, no. 1 (2021): 1–8, 10.1186/s12885-021-08718-3. 34461861 PMC8406897

[5] B. Cutuli , M. Lacroze , J. M. Dilhuydy , et al., “Male Breast Cancer: Results of the Treatments and Prognostic Factors in 397 Cases,” European Journal of Cancer 31, no. 12 (1995): 1960–1964. 10.1016/0959-8049(95)00366-5 8562148

[6] F. Cardoso , J. M. S. Bartlett , L. Slaets , et al., “Characterization of Male Breast Cancer: Results of the EORTC 10085/TBCRC/BIG/NABCG International Male Breast Cancer Program,” Annals of Oncology 29, no. 2 (2018): 405–417. 29092024 10.1093/annonc/mdx651 PMC5834077

[7] F. Wang , X. Shu , I. Meszoely , et al., “Overall Mortality After Diagnosis of Breast Cancer in Men vs Women,” JAMA Oncology 5, no. 11 (2019): 1589. 31536134 10.1001/jamaoncol.2019.2803 PMC6753503

[8] S. Johnston , M. Martin , A. Di Leo , et al., “MONARCH 3 Final PFS: A Randomized Study of Abemaciclib as Initial Therapy for Advanced Breast Cancer,” NPJ Breast Cancer 5, no. 1 (2019): 5515. 10.1038/s41523-018-0097-z PMC6336880 30675515

[9] G. N. Hortobagyi , S. M. Stemmer , H. A. Burris , et al., “Overall Survival With Ribociclib Plus Letrozole in Advanced Breast Cancer,” New England Journal of Medicine 386, no. 10 (2022): 942–950, 10.1056/NEJMoa2114663. 35263519

[10] R. S. Finn , M. Martin , H. S. Rugo , et al., “Palbociclib and Letrozole in Advanced Breast Cancer,” New England Journal of Medicine 375, no. 20 (2016): 1925–1936, 10.1056/NEJMoa1607303. 27959613

[11] S. A. Im , Y. S. Lu , A. Bardia , et al., “Overall Survival With Ribociclib Plus Endocrine Therapy in Breast Cancer,” New England Journal of Medicine 381, no. 4 (2019): 307–316, 10.1056/NEJMoa1903765. 31166679

[12] H. Ç. Yıldırım , E. Mutlu , E. Chalabiyev , et al., “Clinical Outcomes of Cyclin‐Dependent Kinase 4–6 (CDK 4–6) Inhibitors in Patients With Male Breast Cancer: A Multicenter Study,” Breast: Official Journal of the European Society of Mastology 66 (2022): 85–88. 36208540 10.1016/j.breast.2022.09.009 PMC9547301

[13] A. Ring , M. Karuturi , E. N. Smyth , et al., “Characteristics and Outcomes in Cases of US Male Patients With Metastatic Breast Cancer Receiving Abemaciclib in Routine Clinical Practice,” Advances in Therapy 40, no. 5 (2023): 2515–2523. 36995468 10.1007/s12325-023-02471-8 PMC10129913

[14] D. Hansra , S. Jackson , J. Sequeira , R. Vazirani , and R. Alvarez , “Male Patient With Metastatic Stage IV Breast Cancer Achieves Complete Remission on Second Line Abemaciclib, Fulvestrant and Leuprolide: A Case Report,” Molecular and Clinical Oncology 12, no. 2 (2020): 120–125. 31929882 10.3892/mco.2019.1955 PMC6951239

[15] R. Torres , B. Calsina , A. Hermoso , et al., “Abstract 2836: Characterization of the Mechanism of Action for Abemaciclib With Antiestrogen Combined Therapy in Human Breast Cancer Cell Lines,” Cancer Research 76, no. 14_Supplement (2016): 2836.26896281

[16] S. A. Wander , N. O'brien , L. M. Litchfield , et al., “Targeting CDK4 and 6 in Cancer Therapy: Emerging Preclinical Insights Related to Abemaciclib,” Oncologist 27, no. 10 (2022): 811. 35917168 10.1093/oncolo/oyac138 PMC9526495

[17] L. M. Spring , S. A. Wander , F. Andre , B. Moy , N. C. Turner , and A. Bardia , “Cyclin‐Dependent Kinase 4 and 6 Inhibitors for Hormone Receptor‐Positive Breast Cancer: Past, Present, and Future,” Lancet 395, no. 10226 (2020): 817–827. 32145796 10.1016/S0140-6736(20)30165-3

[18] J. L. Blum , C. DiCristo , D. Gordon , et al., “Outcomes of Male Patients With HR+/HER2– Advanced Breast Cancer Receiving Palbociclib in the Real‐World POLARIS Study,” Breast Cancer Research and Treatment 203, no. 3 (2024): 463. 37903899 10.1007/s10549-023-07145-1 PMC10805882

[19] F. Zagouri , T. N. Sergentanis , H. A. Azim , D. Chrysikos , M. A. Dimopoulos , and T. Psaltopoulou , “Aromatase Inhibitors in Male Breast Cancer: A Pooled Analysis,” Breast Cancer Research and Treatment 151, no. 1 (2015): 141–147. 25850534 10.1007/s10549-015-3356-9

[20] C. M. Costelloe , H. H. Chuang , J. E. Madewell , and N. T. Ueno , “Cancer Response Criteria and Bone Metastases: RECIST 1.1, MDA and PERCIST,” Journal of Cancer 1, no. 1 (2010): 80. 20842228 10.7150/jca.1.80 PMC2938069

